# Sorafenib generates microvesicle particles in non-small cell lung cancer

**DOI:** 10.36922/td025110019

**Published:** 2025-06-19

**Authors:** Yevgeniy Gladkiy, Anita Thyagarajan, Morgann Hendrixson, Ravi P. Sahu

**Affiliations:** 1 Boonshoft School of Medicine, Wright State University, Dayton, Ohio, United States of America; 2 Department of Pharmacology and Toxicology, Boonshoft School of Medicine, Wright State University, Dayton, Ohio, United States of America

**Keywords:** Non-small cell lung cancer, Tyrosine kinase inhibitors, Sorafenib, Platelet-activating factor-receptor, Acid sphingomyelinase, Microvesicle particles

## Abstract

Despite the improved clinical outcomes resulting from the use of sorafenib, the development of resistance mechanisms continues to undermine its treatment efficacy. Recent studies have implicated the role of a phospholipid mediator, platelet-activating factor receptor (PAFR) pathway, and extracellular vesicles known as microvesicle particles (MVP) in influencing cellular behavior and the efficacy of therapeutic agents. In this study, we determined the impact of the PAFR pathway and the acid sphingomyelinase (aSMase), which is required for the biogenesis of MVP, on sorafenib-induced effects on lung cancer growth and MVP release. Using A549 and H1299 non-small cell lung cancer (NSCLC) cell lines, we showed that sorafenib treatment reduced cell viability in a dose and time-dependent manner. Notably, sorafenib also enhanced MVP formation in both NSCLC cell lines. This MVP release was significantly attenuated by pharmacologic inhibition of the PAFR pathway through the WEB2086 compound and the aSMase inhibitor, imipramine, indicating the involvement of the PAFR and aSMase in sorafenib-induced MVP biogenesis. Moreover, co-treatment with imipramine enhanced the cytotoxic effects of sorafenib, suggesting that targeting MVP-associated pathways may improve sorafenib response. Collectively, these findings offer mechanistic insight into how sorafenib modulates MVP release and supports the therapeutic potential of combining tyrosine kinase inhibitors with agents that disrupt MVP biogenesis in NSCLC.

## 1. Introduction

Lung cancer is the leading cause of cancer-related mortality in the United States and worldwide.^1^ It is estimated that 234,580 new cases and 125,070 deaths (~20% of all cancer-related deaths) are attributed to lung cancer.^2^ Of the two subtypes, non-small cell lung cancer (NSCLC) accounts for about 80 – 85% of all lung cancer cases.^2^ The management of NSCLC includes chemotherapy, immunotherapy, and targeted treatments that have enhanced survival outcomes, particularly in patients with early-stage or resectable disease.^3^ Notably, advances in immune checkpoint inhibitors and targeted therapies have provided tailored options based on tumor characteristics such as programmed cell death ligand 1 (PD-L1) expression and specific genetic mutations (*i.e*., epidermal growth factor receptor [EGFR] and anaplastic lymphoma kinase) leading to better disease control and prolonged survival.^3^ Despite these advances, emergence of resistance mechanisms remains a significant challenge, which includes on-target mutations, bypass pathways, and histological transformation.^3^

Among targeted therapies, tyrosine kinase inhibitors (TKIs), including sorafenib, have been used to treat NSCLC.^3,4^ Sorafenib, a multikinase inhibitor, has emerged as a promising agent targeting multiple pathways involved in tumor progression, angiogenesis, and resistance mechanisms.^4^ Notably, reactive oxygen species (ROS) generation is one of the key mechanisms through which TKIs induce cytotoxic effects; however, elevated ROS levels activate resistance mechanisms enabling the tumor to evade therapy and continue to grow.^5^ For example, oxidative modifications of EGFR and associated downstream signaling pathways enhance tumor progression and resistance to EGFR TKIs.^6^ These findings highlight the paradoxical nature of ROS in NSCLC therapy. While ROS generation is critical for the effectiveness of TKIs, the adaptive responses of NSCLC cells to oxidative stress underscore the need for combination strategies that both amplify ROS cytotoxicity and inhibit resistance pathways to improve therapeutic outcomes. Among these signaling pathways, ROS non-enzymatically cleaves lipid membranes to produce oxidized glycerophosphocholines (Ox-GPCs) that exhibit platelet-activating factor (PAF) agonistic properties, which mediate angiogenesis, tumor growth, metastasis, and immune modulation.^7–10^ In addition, PAF-like molecules are often upregulated in response to radiation and chemotherapy, exacerbating immune suppression and therapy resistance.^10–13^ Our group has shown that in NSCLC models, both tumor and its environment are modulated by PAF and platelet-activating factor-receptor (PAFR) signaling.^9,14,15^ Moreover, PAFR plays a significant role in vesicular formation and is dependent on pathways such as mitogen-activated protein kinase (MAPK), nuclear factor kappa B (NF-κB), and acid sphingomyelinase (aSMase).^16–19^ Notably, formed vesicles have been shown to contain PAF-like agonists and serve as bioactive molecules.^18–22^

Mounting evidence points to these large extracellular vesicles, also referred to as microvesicle particles (MVP), as critical mediators of treatment resistance, including NSCLC.^23–26^ By sequestering and exporting oncogenic proteins, nucleic acids, and even chemotherapeutic agents, MVP can diminish drug accumulations in tumor cells and modulate the surrounding microenvironment to favor cancer progression.^23,24^ It has been demonstrated that PAF and related lipid mediators can be packaged within MVP, enabling inflammatory and immune-modulating responses that further compromise treatment efficacy.^18,20–22^ These findings underscore that in addition to targeting primary oncogenic pathways, strategies that disrupt MVP release may aid in overcoming drug resistance.

Imipramine, a tricyclic antidepressant, has garnered attention as an effective aSMase inhibitor that disrupts ceramide biosynthesis, a key lipid mediator in MVP formation and NSCLC pathophysiology.^27–32^ By reducing ceramide production, imipramine decreases the budding of vesicles, thus curtailing MVP release.^27,33^ Therefore, imipramine and other sphingolipid-targeted drugs have been of interest as adjunct therapies.^27,34–37^ For example, studies by Irep *et al*.,^32^ have demonstrated enhanced inhibitory effects on cisplatin/etoposide by targeting small extracellular vesicles (also referred to as exosomes) synthesis and trafficking in a small cell lung cancer model. In NSCLC, sorafenib’s efficacy has also been shown to significantly improve with dual-therapy approaches.^4,38–40^ However, no approach to aSMase inhibition, such as with imipramine, has been investigated.

## 2. Materials and methods

### 2.1. Reagents

Culture media was obtained from GE Healthcare Biosciences (Marlborough, MA, USA), with fetal bovine serum (FBS) from Corning (Corning, NY, USA). Penicillin-streptomycin was acquired from Hyclone (Logan, UT, USA) and antibiotic-antimycotic solution was purchased from Gibco (Gaithersburg, MD, USA). The PAFR agonist carbamoyl-PAF (CPAF), the antagonist WEB2086, and the aSMase inhibitor imipramine were all obtained from Cayman Chemicals Co. (Ann Arbor, MI, USA). Sorafenib tosylate was procured from Millipore Sigma (St. Louis, MO, USA). All other reagents were purchased from Sigma-Aldrich (St. Louis, MO, USA).

### 2.2. Cell lines

Human NSCLC lines, A549 and H1299, were used for all experiments as both express PAFR at similar levels.^14^ These cell lines were a kind gift from Dr. Weiwen Long (Department of Biochemistry and Molecular Biology at Wright State University). A549 cells were maintained in F-12K medium supplemented with 10% FBS, 2.5 mL penicillin-streptomycin, 2.5 mL antibiotic-antimycotic, and 15 μL of 2 M magnesium chloride. H1299 cells were grown in RPMI-1640 medium containing 10% FBS, 2.5 mL penicillin-streptomycin, 2.5 mL antibiotic-antimycotic, 2.25 mL of 40% glucose, and 5 mL of 100 mM sodium pyruvate. All cell cultures were maintained at 37°C with 95% humidity and 5% CO_2_.

### 2.3. Cell survival assay

As per our previous reports, sulforhodamine-B (SRB) assay was used to assess cell survival.^14,15^ H1299 and A549 cells were plated into 96-well plates at a seeding density of 5 × 10^3^ cells per well and treated with 0.1% dimethyl sulfoxide (DMSO) as control, or with sorafenib at concentrations ranging from 1 to 16 μM. In separate experiments, sorafenib was used at a concentration of 4 μM, imipramine at 20 μM, and their co-treatment at given concentrations. After 24, 48, and 72 h, cells were fixed with 100 μL of 10% trichloroacetic acid followed by incubation at 4°C for 1 h. Fixed cells were gently rinsed with distilled water three times and stained using 100 μL 0.4% (w/v) SRB (prepared in 1% acetic acid), followed by 15-min incubation at room temperature in the dark. Excess dye was removed by triple rinsing with distilled water containing 1% glacial acetic acid and then allowed to air dry. Bound SRB dye was solubilized using 150 μL of 10 mM Tris base (tris(hydroxymethyl) aminomethane) while placing the plates on a shaker for 10 min. Absorbance was measured at 570 nm using a Synergy H1Mf plate reader. Cell viability for each group was normalized to its respective vehicle-treated control (0.1% DMSO).

### 2.4. MVP isolation and quantification

Isolation and quantification of MVP were performed using methods previously described by our group.^14,18,19^ Briefly, A549 and H1299 cells were grown to approximately 80–90% confluency, after which cultures were rinsed three times with serum-free Hanks’ Balanced Salt Solution (HBSS, Cytiva, USA). Cells were then incubated with 0.1% DMSO for negative control, or 100 nM CPAF and phorbol myristate acetate (PMA) for positive controls, and sorafenib at various concentrations (4, 8, and 16 μM) in HBSS containing 1% free fatty acid. Similarly, combination experiments used pre-treatments with the PAFR antagonist, WEB2086 (10 μM), and imipramine (20 μM) for 1 h, followed by treatment with or without sorafenib (8 μM). After 4 h of incubation, the conditioned medium was centrifuged at 2,000 ×*g* for 20 min at 4°C to remove residual cells and debris. The clarified supernatant was centrifuged at 20,000 ×*g* for 70 min at 4°C to pellet MVP. Pellets were then resuspended with 100 μL of sterile-filtered phosphate-buffered saline (PBS) to prepare for nanoparticle tracking analysis. MVP concentration was assessed using the NanoSight NS300 instrument (Malvern Instruments, UK). MVP counts were normalized with the cell number as per previous reports.^14,18,19^

### 2.5. Statistical analysis

All statistical analyses were conducted using GraphPad Prism software version 10 (GraphPad Software, San Diego, CA, USA). Each *in vitro* experiment was performed independently at least three times using biological replicates. Data were analyzed by unpaired Student’s *t*-test or one-way analysis of variance (ANOVA) with *post hoc* Dunnet’s multiple comparison tests. The *p*<0.05 was considered statistically significant.

## 3. Results

### 3.1. Sorafenib inhibits the survival of NSCLC cell lines in a time- and dose-dependent manner

**Figure 1.**
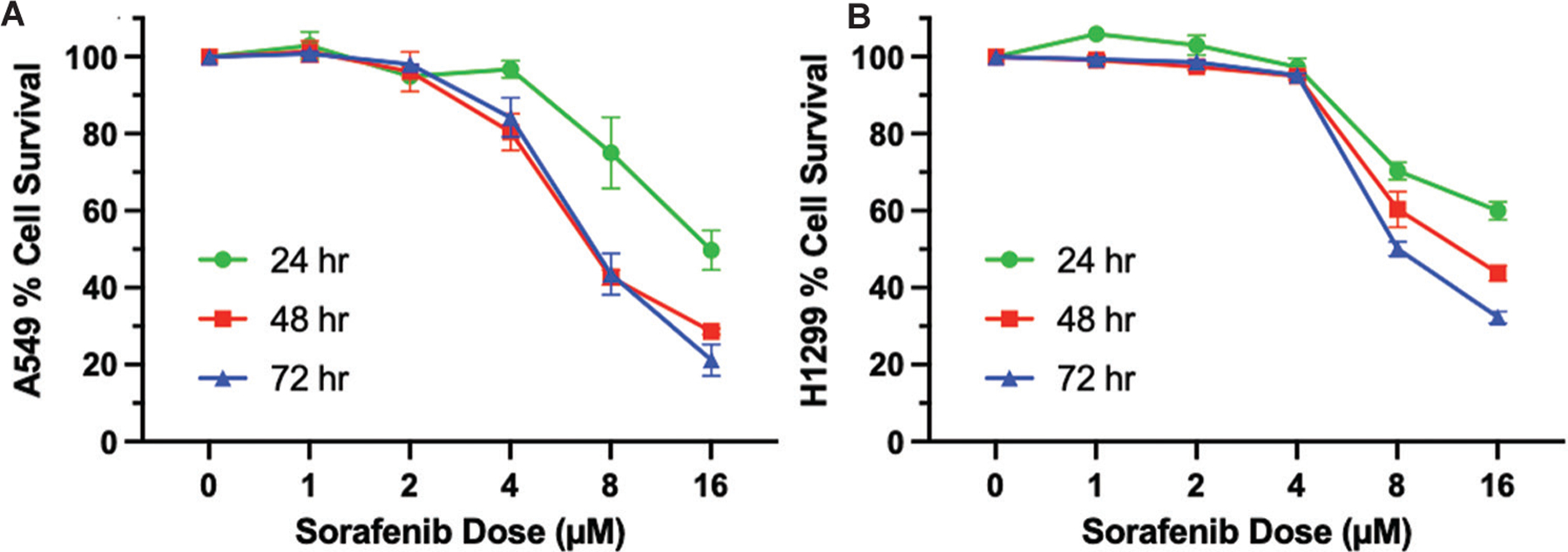
Effects of sorafenib on cell survival. (A) Dose response curve of sorafenib effect on A549 cells. (B) Dose response curve of sorafenib effect on H1299 cell lines. Data are presented as mean ± scanning electron microscope of four independent biological replicates.

Our first studies tested the dose- and time-response effects of sorafenib treatment on the survival of A549 and H1299 NSCLC cell lines through the SRB assay. These cell lines have been widely used as NSCLC models to determine the mechanisms and cellular responses of sorafenib alone or its combination with other agents.^39,41–44^ It was observed that the survival of A549 and H1299 cell lines was inhibited by sorafenib in a dose- and time-dependent manner (Figure 1A and B).

Interestingly, despite both A549 and H1299 cells lacking EGFR mutations and being inherently resistant to EGFR-TKIs,^45^ A549 cells demonstrated greater sensitivity to sorafenib compared to H1299 cells. This observation is consistent with previous report showing that A549 cells, which harbor a *KRAS* G12S mutation, are more susceptible to sorafenib’s effects, likely due to its inhibition of RAF-dependent signaling.^46^ Given that sorafenib also targets vascular endothelial growth factor receptor (VEGFR) and platelet-derived growth factor receptor (PDGFR), its anti-proliferative effect on A549 cells may also involve angiogenic signaling pathways.^47^

Despite being p53-null and KRAS wild-type, H1299 cells also exhibited a significant reduction in cell viability with sorafenib treatment. However, the degree of inhibition was lower than in A549 cells at comparable doses. This suggests that sorafenib’s mechanism of action may be more effective in KRAS-mutant NSCLC models, aligning with findings from previous studies utilizing A549 and PC-9 cells.^38^

### 3.2. PAFR and aSMase pathways mediate sorafenib-induced MVP release

**Figure 2.**
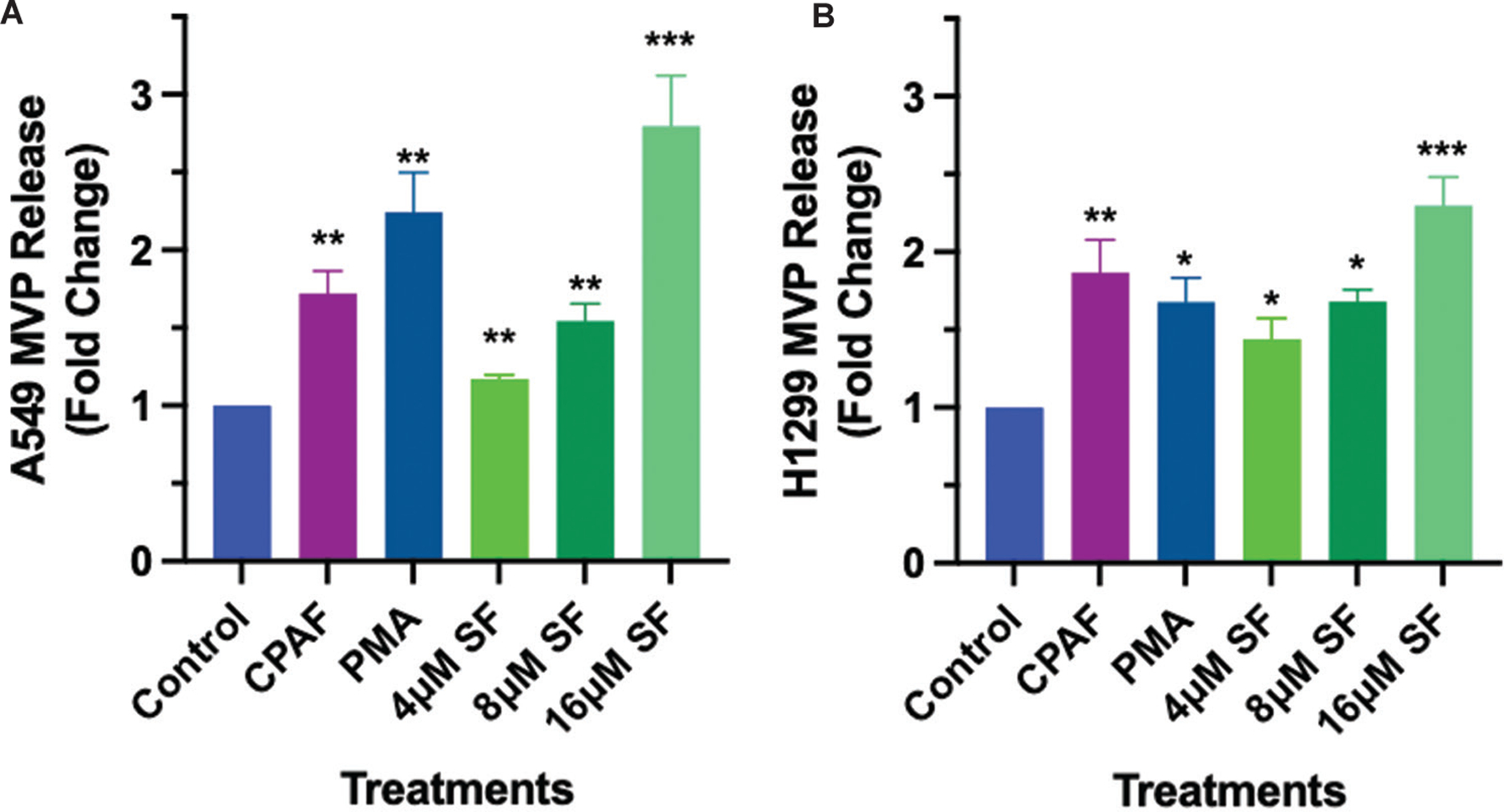
Dose-response effect of sorafenib treatment on MVP release. A549 (A) and H1299 (B) cell lines were treated with vehicle (0.1% DMSO), CPAF (100 nM), PMA (100 nM), and various doses of sorafenib. After 4 h of incubation, MVP extraction and analyses were performed. Data are presented as mean ± scanning electron microscope of three independent biological replicates, normalized per 1 × 10^6^ cells. Statistically significant differences were observed between control and other groups. Notes: *p<0.05, **p<0.01, ***p<0.001. Abbreviations: CPAF: Carbamoyl-platelet-activating factor; MVP: Microvesicle particle; PMA: Phorbol myristate acetate; SF: Sorafenib.

Given that exposure to EGFR-TKIs induces MVP release,^14^ which have been shown to carry PAF agonists and serve as mediator of PAFR-induced effect,^18,19^ our next studies evaluated if sorafenib treatment can induce MVP release. Furthermore, as MVP release is an earlier event, which significantly peaks at 4 – 8 h time points,^14^ and could impact tumor cell behavior in responses to therapeutic agents, we tested three different doses (4, 8, and 16 μM) of sorafenib from the cell viability assay shown in Figure 1A and 1B, that resulted in differential cytotoxic response. To that end, A549 and H1299 cell lines were separately treated with vehicle (0.1% DMSO) as a negative control, CPAF (a known PAFR agonist, 100 nM) and PMA (PAFR-independent agonist, 100 nM) as positive controls, and various doses of sorafenib. After 4 h, we extracted and analyzed MVP as per our published reports.^14,18,19^ The data demonstrated that sorafenib induces MVP release from both cell lines in a dose-dependent manner as compared to vehicle control (Figure 2A and B). In addition, we found that sorafenib-mediated MVP release was comparable to CPAF and PMA treatments (Figure 2A and B).

**Figure 3.**
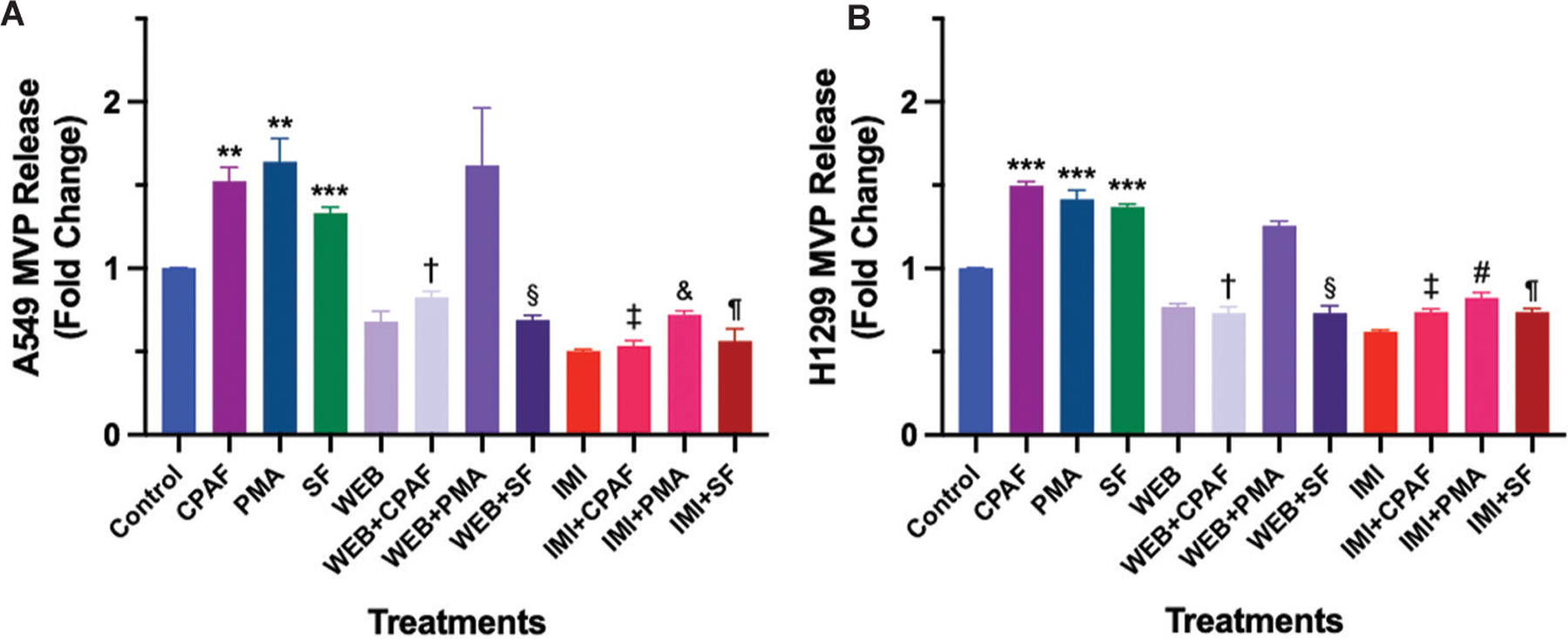
Effects of PAFR antagonist and aSMase inhibitor on sorafenib-induced MVP release. A549 (A) and H1299 (B) cells were pre-treated with WEB2086 (a PAFR antagonist, 10 μM, 1 h) or imipramine (an aSMase inhibitor, 20 μM, 1 h) followed by the treatments with or without CPAF (100 nM), PMA (100 nM), or sorafenib (8 μM). These cell lines were also treated with vehicle (0.1% DMSO), WEB2086 (10 μM) and imipramine (20 μM) alone. After 4 h of incubation, MVP were isolated and analyzed. Data are presented as mean ± scanning electron microscope of three independent biological replicates, normalized per 1 × 10^6^ cells. The statistically significant differences were observed between control and CPAF, PMA, and sorafenib alone groups; CPAF and WEB+CPAF; SF and WEB+SF; CPAF and IMI+CPAF; PMA and IMI + PMA; and SF and IMI + SF. Notes: **p<0.01, ***p<0.001 compared with control; ^†^*p*<0.001 compared with CPAF; ^§^*p*<0.001 compared with SF; ^‡^*p*<0.001 compared with CPAF; ^&^*p*<0.05 compared with PMA; ^#^*p*<0.001 compared with PMA; ^¶^*p*<0.001 compared with SF. Abbreviations: aSMase: Acid sphingomyelinase; CPAF: Carbamoyl-platelet-activating factor; IMI: Imipramine; MVP: Microvesicle particles; PAFR: Platelet-activating factor-receptor; PMA: Phorbol myristate acetate; SF: Sorafenib; WEB: WEB2086.

As PAFR activation mediates MVP release, and the aSMase is a key mediator of MVP biogenesis,^14^ our next studies determined the underlying mechanisms, particularly, the roles of the PAFR signaling and an aSMase using the optimal dose (8 μM) of sorafenib. To that end, A549 and H1299 cell lines were pre-treated with a well-known PAFR antagonist, WEB2086 (10 μM),^14^ or an aSMase inhibitor, imipramine (20 μM),^14^ followed by the treatments with or without CPAF, PMA, or sorafenib. After 4 h, we extracted and analyzed MVP. Our studies demonstrated that the WEB2086 compound significantly blocked CPAF and sorafenib-induced, but not PMA-induced MVP release in both the cell lines (Figure 3A and B), indicating the involvement of the PAFR signaling in MVP release. On the other hand, imipramine significantly blocked CPAF-, PMA-, and sorafenib-induced MVP release, indicating that involvement of an aSMase in MVP release (Figure 3A and B). These data also indicate that regardless of the nature of the stimuli used, inhibiting aSMase blocks MVP release. These data are consistent with our previous findings,^14,18,19^ demonstrating that other ROS-generating stimuli induce MVP release in a PAFR and aSMase-dependent manner.

### 3.3. Imipramine enhances the antiproliferative effect of sorafenib

**Figure 4.**
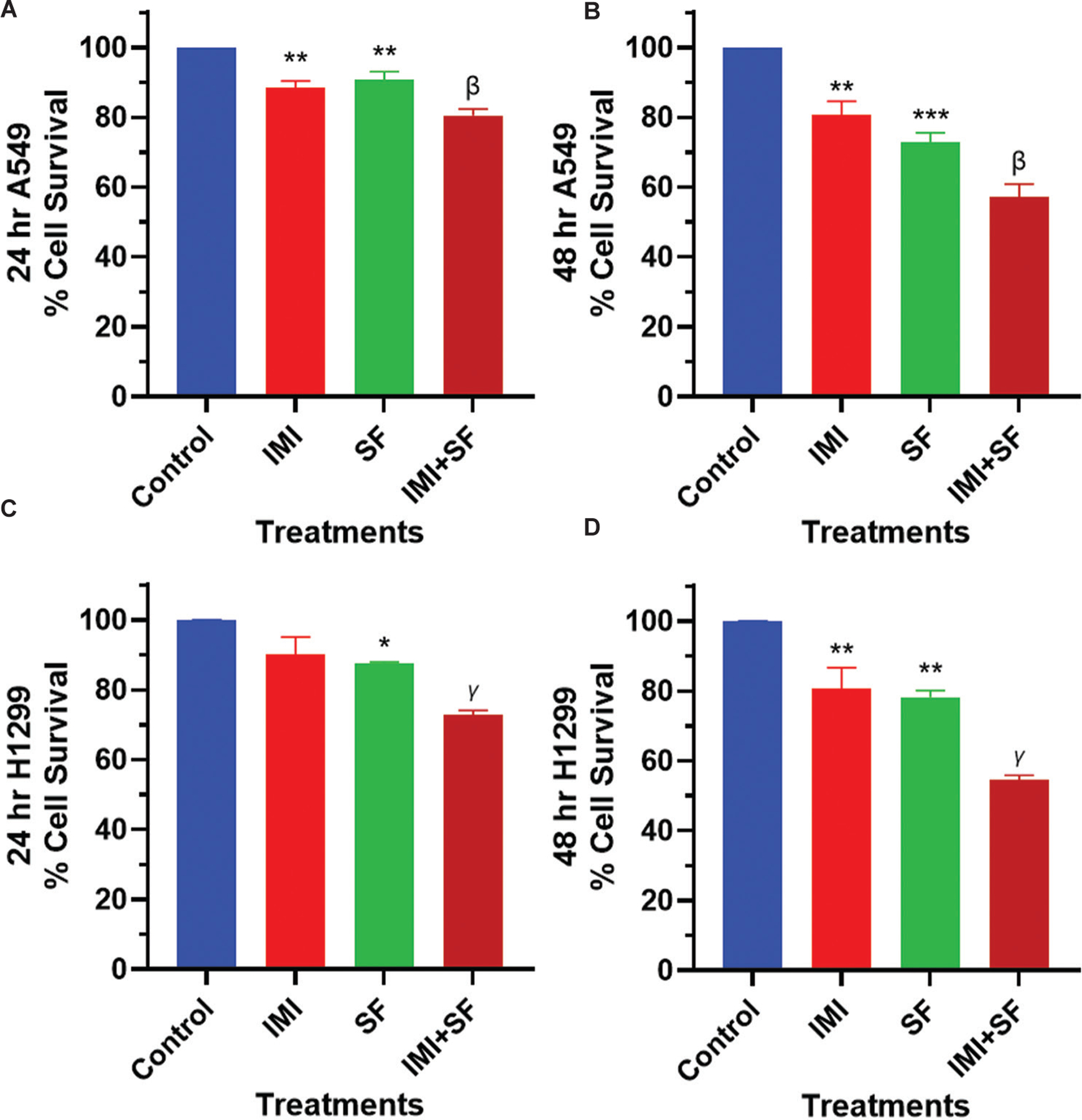
Effect of an aSMase inhibitor on sorafenib cytotoxicity. A549 cells (A, B) and H1299 cells (C, D) were pre-treated with imipramine (an aSMase inhibitor, 20 μM for 1 h) followed by treatment with or without sorafenib (4 μM). After 24 and 48 h, cell viability was assessed through sulforhodamine-B assay. Data are presented as mean ± scanning electron microscope of three independent biological replicates. Statistically significant differences were observed between control and imipramine or sorafenib alone, as well as sorafenib and sorafenib with imipramine co-treatment. Notes: **p<0.01, ***p<0.001 compared with control; ^β^*p*<0.05 compared with SF; ^γ^*p*<0.001 compared with SF. Abbreviations: aSMase: Acid sphingomyelinase; IMI: Imipramine; SF: Sorafenib.

Given that aSMase inhibitors block MVP release and have been evaluated in cancer patients,^34,48^ the next studies tested if blocking aSMase could increase the efficacy of sorafenib. To evaluate the synergy of an aSMase inhibitor on sorafenib-mediated growth inhibition in NSCLC cells, A549 and H1299 cells were pre-treated with imipramine (20 μM for 1 h),^14^ followed by treatment with or without sorafenib at a lower concentration (4 μM), consistent with prior studies utilizing lower micromolar concentrations of sorafenib in combination strategies.^41,49^ The cell survival was assessed using the SRB assay at 24- and 48-h time points. As shown in Figure 4A–D, imipramine enhanced the cytotoxic effect of sorafenib resulting in a significant reduction in cell viability compared to sorafenib monotherapy. We also noticed a modest but significant inhibition of cell viability by imipramine alone at the 48-h time point (Figure 4B and D), indicating a chemopreventive ability of this repurposed drug, providing a rationale for it to be explored in combination with other therapeutic agents.

**Figure 5.**
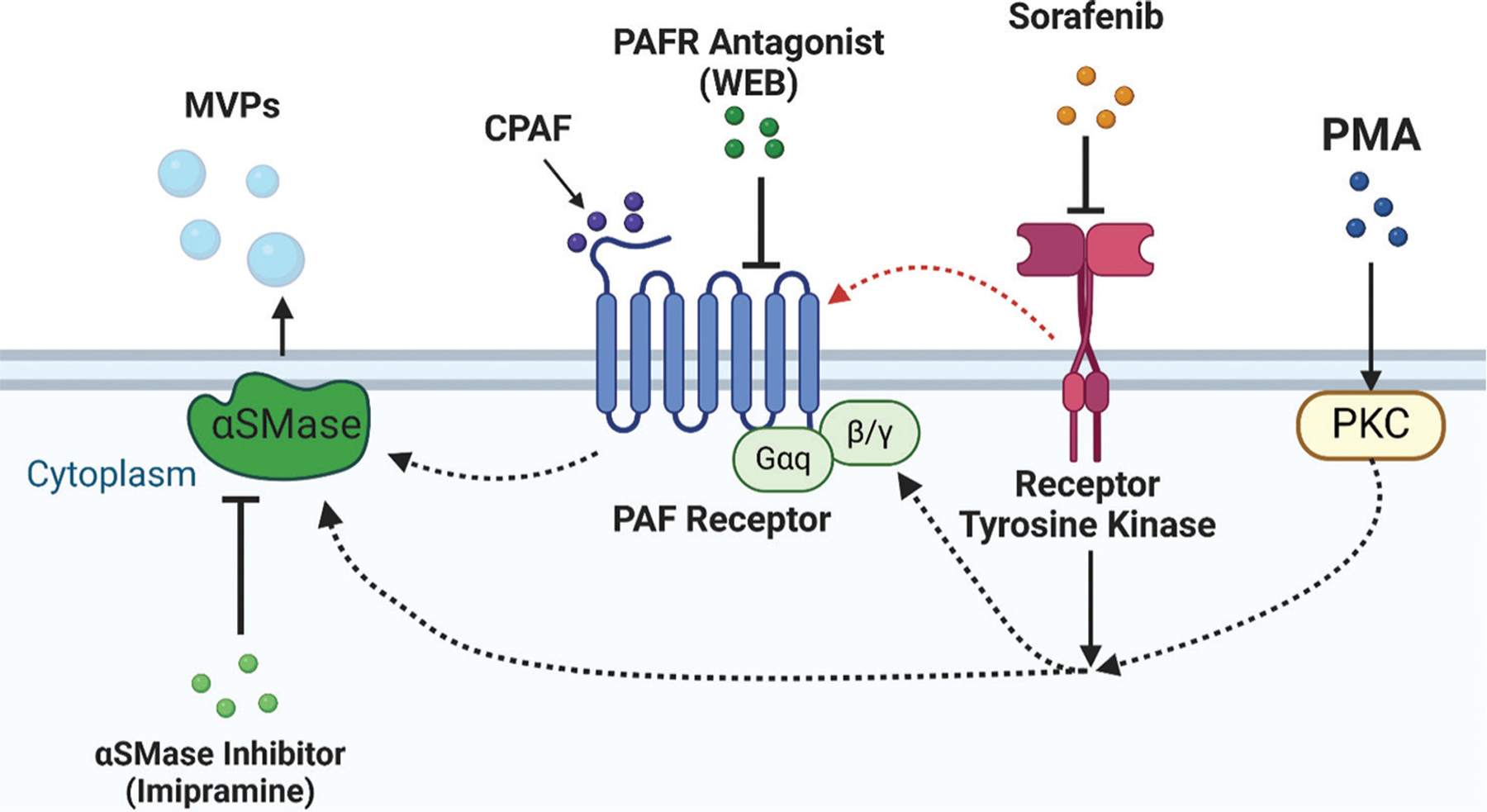
Schematic representation of PAFR and aSMase-dependent MVP release. Created in BioRender. Gladkiy, Y. (2025) https://BioRender.com/ a16u165. Abbreviations: aSMase: Acid sphingomyelinase; CPAF: Carbamoyl-platelet-activating factor; MVP: Microvesicle particles; PAFR: Platelet-activating factor-receptor; PKC: Protein kinase C; PMA: Phorbol myristate acetate; WEB: WEB2086.

Taken together, these results suggest that imipramine enhances the antiproliferative effects of sorafenib, through its ability to inhibit aSMase-mediated pathways, thereby reducing ceramide production and MVP release, as shown in Figure 5. These findings highlight the potential implication of imipramine to enhance the efficacy of sorafenib in NSCLC.

## 4. Discussion

As NSCLC continues to pose challenges,^1–3^ sorafenib, a multikinase inhibitor, has demonstrated variable antitumor effects in NSCLC models by targeting multiple signaling pathways, including those associated with angiogenesis and ROS generation.^3,4^ Although ROS can mediate cytotoxicity in tumors, elevated levels of ROS may paradoxically enhance survival and promote resistance through compensatory pathways.^5,6^ Consequently, combination approaches that both exploit sorafenib’s cytotoxic potential and suppress parallel pro-survival pathway have garnered significant attention in efforts to improve NSCLC outcomes.^26^

A growing body of evidence implicates MVP as a mediator of therapy resistance, tumor progression, and immune evasion in multiple cancer models, including NSCLC.^23,25^ By encapsulating pro-survival factors, oncogenic proteins, or even chemotherapeutic agents, MVP can attenuate the intracellular accumulation of drugs and facilitate communications within the tumor microenvironment that favor cancer cell survival.^23,24^ Our findings indicate that sorafenib treatment increases MVP release in NSCLC cell lines, aligning with prior work demonstrating that other anticancer agents also elevate MVP shedding.^14,19^ This phenomenon may represent an adaptive mechanism by which cancer cells reduce intracellular drug toxicity and exchange signals conducive to tumor growth.

Notably, PAFR signaling and aSMase activity both emerged as critical players in mediating MVP generation. In line with previous reports, PAFR activation appears to drive MVP release across various cancers, including NSCLC.^9,14,18^ Similarly, aSMase catalyzes the hydrolysis of sphingomyelin to ceramide, a lipid known to promote membrane budding and MVP formation.^27,28,33^ Our data confirm that pharmacological blockade of PAFR (via WEB2086) or inhibition of aSMase (via imipramine) substantially diminishes sorafenib-induced MVP release in NSCLC cell lines. These results underscore a therapeutic opportunity, indicating that targeting the MVP production pathways may enhance the efficacy of established anticancer drugs by reducing the vesicular export of survival signals and other resistance factors.

Importantly, imipramine, a tricyclic antidepressant, has garnered attention for its potent aSMase-inhibiting properties, restricting ceramide-dependent MVP biogenesis.^27^ In our experiments, co-treatment with imipramine significantly attenuated MVP generation triggered by sorafenib, reinforcing the concept that MVP blockade might resensitize tumor cells to therapy. As PAFR-mediated MVP release is dependent on pathways, such as MAPK and NF-κB, which crosstalk with aSMase, and sorafenib targets MAPK and NF-κB pathways,^16–19,50^ we anticipate that these downstream signaling cascades could be involved in mediating sorafenib-induced MVP release. Notably, imipramine also enhanced the antiproliferative effect of sorafenib on both A549 and H1299 cell lines, echoing prior studies in other lung cancer models where combined extracellular vesicle inhibition and chemotherapy improved therapeutic outcomes.^32^ Given that MVPs contain PAF-like agonists and serve as bioactive molecules,^18–22^ these findings point to a potential synergy wherein sorafenib disrupts key oncogenic pathways, while imipramine obstructs MVP-mediated drug efflux and paracrine signaling. Such a combination strategy may thus counteract adaptive resistance more effectively than either agent alone.

Sorafenib has previously been shown to exhibit synergistic or additive effects when combined with other agents, including gemcitabine, pemetrexed, and erlotinib.^38–40,51,52^ In each case, multi-target inhibition or blockade of complementary pathways amplified the overall antitumor response. Our data on the sorafenib–imipramine partnership extend this notion by focusing on MVP-mediated resistance, highlighting a novel mechanism that can be exploited to improve therapeutic outcomes (Figure 5). Although further *in vivo* investigation is warranted, these findings contribute to the broader literature advocating for rationally designed combination regimens in NSCLC.

Despite these promising insights, several limitations must be addressed. First, our work is primarily based on *in vitro* models using A549 and H1299 cell lines, which do not fully represent the complexities of human tumors. Second, the specific downstream signaling events by which sorafenib-induced MVP promotes resistance remain to be fully characterized. Third, while imipramine has demonstrated its efficacy as an aSMase inhibitor, its clinical repurposing requires careful consideration of known dose-dependent toxicities, including anticholinergic side effects.^27,53^ Further research may benefit from evaluating more selective aSMase inhibitors and novel drug delivery systems to improve safety profiles and efficacy.^34^ Finally, the optimal dosing, timing, and safety profile for combining imipramine with sorafenib have yet to be delineated, highlighting the need for rigorous *in vivo* studies and ultimately, clinical trials. Identifying patients most likely to benefit from such a combination – potentially through biomarkers such as high basal MVP release or elevated aSMase expression – also represents an important area for future research.^54,55^

## 5. Conclusion

Overall, our findings underscore the importance of targeting MVP production to overcome adaptive resistance in NSCLC. By combining sorafenib with imipramine, our studies demonstrated successfully reduced MVP release and enhanced sorafenib’s cytotoxic activity in NSCLC cells. These observations build on accumulating evidence that MVP-focused interventions can potentiate the efficacy of conventional and targeted therapies. Going forward, additional *in vivo* validation and clinical exploration are warranted to determine whether this dual-targeting strategy can translate into improved outcomes for patients with NSCLC.

## Data Availability

All datasets generated for this study are available from the corresponding authors on reasonable request.
